# Physicians’ views on the usefulness and feasibility of identifying and disclosing patients’ last phase of life: a focus group study

**DOI:** 10.1136/bmjspcare-2020-002764

**Published:** 2021-02-22

**Authors:** Catherine Owusuaa, Irene van Beelen, Agnes van der Heide, Carin C D van der Rijt

**Affiliations:** 1 Medical Oncology, Erasmus MC, Rotterdam, Zuid-Holland, The Netherlands; 2 Department of Public Health, Erasmus MC, Rotterdam, Zuid-Holland, The Netherlands

**Keywords:** cancer, chronic obstructive pulmonary disease, communication, prognosis

## Abstract

**Objectives:**

Accurate assessment that a patient is in the last phase of life is a prerequisite for timely initiation of palliative care in patients with a life-limiting disease, such as advanced cancer or advanced organ failure. Several palliative care quality standards recommend the surprise question (SQ) to identify those patients. Little is known about physicians’ views on identifying and disclosing the last phase of life of patients with different illness trajectories.

**Methods:**

Data from two focus groups were analysed using thematic analysis with a phenomenological approach.

**Results:**

Fifteen medical specialists and general practitioners participated. Participants thought prediction of patients’ last phase of life, i.e. expected death within 1 year, is important. They seemed to find that prediction is more difficult in patients with advanced organ failure compared with cancer. The SQ was considered a useful prognostic tool; its use is facilitated by its simplicity but hampered by its subjective character. The medical specialist was considered mainly responsible for prognosticating and gradually disclosing the last phase. Participants’ reluctance to such disclosure was related to uncertainty around prognostication, concerns about depriving patients of hope, affecting the physician–patient relationship, or a lack of time or availability of palliative care services.

**Conclusions:**

Physicians consider the assessment of patients’ last phase of life important and support use of the SQ in patients with different illness trajectories. However, barriers in disclosing expected death are prognostic uncertainty, possible deprivation of hope, physician–patient relationship, and lack of time or palliative care services. Future studies should examine patients’ preferences for those discussions.

**Key messages**

**What was already known?**

- Accurate assessment that a patient is in the last phase of life is a prerequisite for timely initiation of palliative care in patients with a life-limiting disease.
- Several quality standards recommend the surprise question (SQ) to identify patients who may need palliative care.

**What are the new findings?**

- Physicians consider prognostication important because it helps them to think about treatment goals for their patients.
- Physicians are hesitant to open the discussion with patients, because of prognostic uncertainty and unknown consequences for patients, the physician–patient relationship, potential deprivation of hope, and lack of time or palliative care services.
- Physicians support use of the SQ, which could be used more routinely as a means to identify patients in their last phase of life, rather than to accurately predict death.

**What is their significance?**

- Future studies should explore patients’ preferences for disclosing their last phase of life.

##  Background

 In the last phase of life, goals of care need to be realigned with patients’ needs and preferences. Furthermore, patients may experience multiple symptoms for which they need palliative treatment.^1 2^ Timely initiation of advance care planning, a process of discussing patient’s preferences and goals for medical treatment and care in the last phase of life, is essential for providing adequate patient care.^3^

Accurate assessment that a patient is in the last phase of life is a prerequisite for timely initiation of palliative care.^4^ Various international frameworks use a life expectancy of 1 year or less as a criterion to start palliative care. However, the identification of the last phase of life is hampered by variance in disease trajectories. Patients with cancer may undergo significant functional decline in the last months or weeks before death, whereas patients with organ failure, such as chronic obstructive pulmonary disease (COPD), may experience multiple acute exacerbations with ultimate physical decline during a period of one to several years.^5^ The surprise question (SQ)*—Would you be surprised if this patient were to die in the next year?*—is recommended as a screening tool to identify patients with life-limiting diseases who may be in the last phase of life.^6^ The SQ has been adopted in several quality standards worldwide to facilitate palliative care and advance care planning, such as the Gold Standards Framework and the Netherlands Quality Framework for Palliative Care.^7 8^ Studies about the SQ have mainly focused on its accuracy to predict death within one year, especially in patients with cancer or end-stage renal failure, and to a lesser extent on physicians’ experiences with and appreciation of using the SQ for patients with different disease trajectories.^6 9^ We therefore conducted a focus group study with physicians to investigate their views on the usefulness and feasibility of identifying and disclosing patients’ last phase of life. In this study, the last phase was defined as expected death within one year, which is also the timeframe generally used for the surprise question.

## Methods

### Sample

Physicians attending patients with cancer or COPD in the last year of life were included. There were no exclusion criteria. Written invitations were sent to the multidisciplinary oncology boards of eight hospitals to recruit one or two participants from each hospital. Further, using the snowball method through the multidisciplinary oncology boards, we aimed to recruit one pulmonologist from each hospital, specialists in the elderly care from different nursing homes and general practitioners (GPs) from different general practices. Our target sample size was 20, including 16 hospital physicians and four specialists in elderly care or general practice.

### Procedures

Two focus group meetings were held, each lasting 120 min because that seemed a sufficient and acceptable time to discuss the topics as planned. Prior to the meetings, participants were requested to fill out a short questionnaire on their discipline, patient population and acquaintance with the SQ (box 1). The SQ was framed as ‘*Would you be surprised if this patient were to die in the next year?*’. Additional information on the study was provided in the invitation email. The focus group discussions were facilitated by a set of statements on prognostication and the SQ (box 2). These statements were composed by the research team based on our experiences of commonly expressed opinions by physicians about the last phaseof life. One moderator (CCDvdR) and two observers (CO and AvdH) attended both meetings. Under Dutch Law, studies like this are waived from review by a research ethics committee. We considered participants’ agreement to partake in the focus groups as consent for the study. Participants explicitly consented to the focus group being audiotaped.

Box 1 **Questions of online questionnaire**

1. What is your function?
2. Which patients do you attend?
3. Do you think it is generally useful to identify a patient’s last phase of life?
4. Can you generally predict when a patient with cancer or chronic obstructive pulmonary disease has 1 year or less to live?
5. Are you acquainted with the surprise question?

Box 2 **Statements presented during the focus groups**

1. The surprise question is a good and useful method to facilitate prognostication of the last phase of life.
2. Prognostication of the last phase of life is the task of the treating medical specialist.
3. The accurate estimation of the patient’s life expectancy is mainly a matter of clinical experience.
4. After prognosticating the final phase of life, the medical specialist must transfer the management of the care to the general practitioner.
5. The application of prognostic tool for the last phase of life makes discussion about that phase easier for the physician.
6. The application of a prognostic tool to identify the last phase of life makes care impersonal.
7. Prognostication of the last phase of life should be delayed in most situations in order to maintain hope for the patient.

### Data analysis

The audiotaped focus group discussions were transcribed verbatim, removing names of the participants. Based on the use of statements during the focusgroup discussions, we expected to find certain themes in the data analysis. Therefore, data were analysed in three phases using thematic analysis with a phenomenological approach.^10^ First, two researchers (CO and IvB) read the transcripts independently and identified important themes. They compared their preliminary themes and made a preliminary coding tree, which was discussed within the research team (AvdH and CCDvdR), until consensus was reached. Second, one researcher (CO) re-read and coded the transcripts using the coding tree, which was further evaluated together with the second researcher (IvB). Lastly, the researchers integrated the identified themes to a new set consisting of the most relevant themes (online supplemental figure 1).

Theme-related quotes from the focus group meetings to illustrate the findings were selected by one researcher (CO) and approved by the research team. All data were analysed anonymously. The validity of the findings was tested through member checks by asking all participants to review a summary of the findings, which did not result in adaptations. The manuscript was structured using the Standards for Reporting Qualitative Research guideline.^11^

## Results

Fifteen of the 16 physicians who completed the questionnaire (box 1) participated in one of the two focus group meetings: 7 oncologists, 3 GPs, 2 specialists in elderly care, 2 pulmonologists and 1 pain specialist with a background in anesthesiology. There were ten female and five male participants. One elderly care specialist could not attend the focus group meeting. All participants attended patients with cancer and nine participants also attended patients with COPD. The overall themes and subthemes that were identified from the thematic analysis are summarized below (SupplementaryFigure 1).

### Importance of predicting the last phase of life

According to the participating physicians, prediction of the last phase of life is important, its main purpose being to enable timely provision of tailored palliative care to patients. By identifying patients who are expected to die within one year, physicians and care teams can facilitate the proactive evaluation of medical treatment and care in relation to patients’ preferences for the last phase of life.

Well, yes, predicting the last phase of life forces you as a treatment team to not only look at the next available treatment line, but to also critically look at its benefits for a patient who may not have long to live. It is important for physicians to do so because patients might only be focused on that next treatment line. (**Participant 5, medical oncologist**)

### Differences and difficulties in predicting the last phase of life for patients with cancer or COPD

When predicting the last phase of life in patients with cancer or COPD, participants rely mainly on their clinical experience and on their knowledge regarding the patient’s disease stage or tumour type. For example, they can base their predictions on a patient’s performance status or on the disease course, for example, the incidence of exacerbations for patients with COPD or the response on antitumor therapy for patients with cancer. Some participants found it difficult to predict the last phase of life for patients with tumour types for which multiple lines of systemic therapy are available (eg, breast cancer), because expected death may only become evident in case of an acute deterioration after exhausting those treatment lines. Participants who attended patients with cancer as well as patients with COPD found it more difficult to predict the last phase of life in patients with COPD than in patients with cancer, although for some types of cancer it can be particularly difficult as well.

I think the years of experience have made me better in prognostication, even in situations where the trajectory is different from what we expected. It is never easy and never 100% though. (**Participant 15, medical oncologist**)

With breast cancer, it remains difficult because people can live another 10 years with only a few skeletal metastases. (**Participant 13, medical oncologist**)

### Application of the SQ and its facilitators

Most participants thought the SQ is a useful tool to support the identification of patients’ last phase of life. They are typically triggered to use the SQ when they notice significant deterioration of a patient’s condition. Facilitators for the use of the SQ are that it is a simple question, clearly formulated and directly raises awareness about a patient’s last phase of life. Additionally, the SQ is recognisable and, therefore, applicable for patients with various chronic diseases. Physicians base their response to the SQ on a combination of intuition, and patient and disease characteristics.

You do have a certain idea of a patient and you wonder, “I am curious if he will make it”. It is a gut feeling, whether you will say yes or no. (**Participant 12, general practitioner**)

Well, we have been discussing the surprise question extensively at the department this afternoon. I think that the surprise question itself, however subjective it may be, is not so bad in all its simplicity. (**Participant 8, pain specialist**)

### Use of other tools to predict the last phase of life

Participants were not acquainted with other tools than the SQ to predict the last phase of life. Participants’ opinions were divided on whether it is preferable to use one’s own subjective clinical judgement or an objective prognostic tool that combines clinical factors. They thought a prognostic tool may give more accurate predictions than subjective judgement, but a tool including clinical factors would probably also require extra time and effort to complete.

If you would have tools to estimate it [the last phase of life] more reliably, that would help. However, I am very curious if that is possible. (**Participant 15, medical oncologist**)

### Expected tasks from different physicians

All participants agreed that the treating physician, usually the medical specialist, should be responsible for prognostication and disclosure of a patient’s last phase of life. Although the GP could also play a role, the medical specialist knows best when disease-modifying treatment options for the patient are exhausted, and thus has more insight whether or not the patient’s death is expected within one year. Participating GPs emphasised that the medical specialist should inform the patient’s GP timely about the exhaustion of treatment and the last phase of life. Thereafter, the GP should gradually take more responsibility in the further exploring and realising patients’ preferences for end-of-life care.

I appreciate clear prognostication from the medical specialist. For example, that there are no more treatments available for the patient, because it is difficult for me to remain knowledgeable of all treatment options. (**Participant 4, general practitioner**)

### Timing of disclosure of the last phase of life

All participants thought that acknowledgement of a patient’s last phase of life is important for the initiation of a discussion with patients about their preferences and needs for medical care in the last phase of life. Most participants thought it is useful and feasible to start this discussion early, that is, about 1 year before a patient’s expected death. This timing may then provide patients and relatives with sufficient time to prepare for the last stage of life and make all necessary arrangements. Some participants, however, thought that 1 year might be too early to initiate those discussions; they preferred to open such a discussion in the period ‘during which palliative care is actually required’, the period ‘during which maintaining quality of life outweighs prolonging life’ or ‘in the last 6 months of life’. Physicians should disclose information about a patient’s last phase of life gradually, preferably during multiple conversations, because that gives the patient the opportunity to process the information and to think about preferences for care. Some participants link the timing of those discussions to a significant deterioration of the patient’s disease (eg, progression of metastases or acute COPD exacerbation), or to the discussion of preferences about resuscitation. Other participants thought that disclosing the last phase of life during those moments might increase the patient’s anxiety or panic.

The period of one year has something arbitrary. A trajectory of 1 year is maybe meaningful because the patient can get used to the last phase and make necessary arrangements. (**Participant 1, pulmonologist**)

The last phase of life is an artificial border you draw for yourself. That border, whether 6 months or 1 year, has a different value for each patient. (**Participant 3, medical oncologist**)

It is much harder for patients with COPD who are in acute situations admitted to the hospital, but feel perfectly fine when they are discharged and at home. It is then more difficult to talk about such serious topics [such as death]. My experience with COPD patients is that there is a lot of fear and panic. (**Participant 11, pulmonologist**)

### Inaccurate predictions and disclosures

Participants mentioned several barriers for the disclosure of the last phase of life to patients. First, some participants were concerned about a false prediction of expected death. They found the SQ to be subjective and difficult to answer. Wrong predictions can emotionally harm patients who strictly hang on to those predictions. A few participants mentioned that they had become more reluctant with their predictions due to experiences with patients whose diseases had followed another course than expected.

In all those years, I have sent five people home and said, “You will die within a few days”. All five people were still alive after a year. So you can make huge mistakes. (**Participant 2, medical oncologist**)

### Deprivation of hope

Participants feared that full disclosure of the last phase of life might deprive the patient of hope, especially in patients for whom it is important to maintain hope until the end. Additionally, they were concerned that discussion of the last phase of life may trigger fear in patients or let patients think that the physician is giving up on them. Therefore, they believed that their answer to the SQ should not be disclosed to the patient, unless it is clear that the patient appreciates such disclosure and can cope with it.

Of course, there are always several sides to take into account. Look, you are talking about hope. You also regularly see that people have false hope until the very last chemotherapy, because both the doctor and the patient do not want to talk about the patient’s last phase of life. (**Participant 8, pain specialist**)

I do not want to invoke a lot of fear because of my answer to the surprise question. I do not want to take away hope from patients by telling them they have one year to live, while that could be five years. (**Participant 2, medical oncologist**)

### Impact of physician–patient relationship

Some participants found it difficult to accept the last phase of life of patients with whom they have an established and good physician–patient relationship, or fear that discussions about patients’ last phase of life may affect that relationship. On the other hand, a good physician–patient relationship sometimes makes it easier to initiate the discussion about expected death.

The more you have a relationship with a patient, the more you do not want to see the end coming. That is a major pitfall. (**Participant 15, medical oncologist**)

### Availability of palliative care services and time

Participants felt reluctant to use the SQ and to discuss the last phase of life due to concerns about a lack of palliative care services. Not all hospitals have specialised palliative care teams that can support patients in the last phase of life. Additionally, lack of time during an outpatient consultation makes the initiation of those discussions difficult.

However, the most difficult thing is to start that conversation [about the last phase of life]. I do not think it gets easier. That is also because I do not have a checklist for it and I have [limited] time at the outpatient clinic. (**Participant 5, medical oncologist**)

## Discussion

In this focus group study, we found that physicians consider it important and useful to prognosticate a patient’s last phase of life. In doing so, physicians are enabled to timely assess patients’ preferences for medical treatment and care in the last phase of life. The simply formulated SQ is considered a useful prognostic tool to facilitate prognostication. However, the assumed subjective character of the SQ may hamper its use.^12 13^ Clinical experience with patient and disease-related clinical factors are also facilitators of prognostication. Some studies have indeed found that clinical experience is associated with more accurate predictions of the last phase of life, but other studies found no such associations.^14^,^16^

We found that physicians supported the disclosure of the last phase of life (i.e. expected death within one year) as recommended in quality standards for palliative care,^7 8^ but they also stressed the importance of a gradual disclosure. Furthermore, the primary responsible physician, typically the treating medical specialist, should initiate communication about the last phase of life with patients. However, apart from linking those discussions to moments of significant deterioration in patient’s health, little is known about the best way in which the last phase of life and patient’s wishes and preferences may be discussed. Generally, physicians found the disclosure of the last phase of life difficult, as also supported by previous evidence.^17^ Physicians may find it difficult to initiate such discussions due to uncertainty about prognostication and unknown consequences for patients. They are especially uncertain about predicting death in patients with COPD, which is because organ failure often has a rather fluctuating disease trajectory. Moreover, physicians may be better in predicting death in a late stage of disease.^18^,^20^ Other barriers to initiate discussions about the last phase of life are concerns about depriving patients of hope, concerns about affecting the physician–patient relationship, having insufficient time for a complex conversation, or lacking palliative care services to provide supportive care in response to the assessment and disclosure of a patient’s limited life expectancy. Future studies should explore patients’ preferences for discussing the last phase of life to better address these perceived barriers and difficulties.

Key strengths of our study were the inclusion of a multidisciplinary group of medical specialists and GPs and the participation of physicians with varying expertise. However, several limitations should also be mentioned. First, most participants had affinity with palliative care, which may have caused some level of bias in experiences and views. Second, the number of participating pulmonologists was rather low. Therefore, this study might not have reached data saturation for this group. Third, we did not include the opinions of nonphysician health providers, e.g. nurses. Lastly, we did not gather demographic information about the participants, such as age or work experience in palliative care. Such characteristics might have influenced the views and opinions of the participants.

## Conclusions

Our findings show that physicians consider prognostication as important because it helps them to think about treatment goals for their patients. However, doctors are also hesitant to open the discussion with patients, because of prognostic uncertainty and unknown consequences for patients, deprivation of hope, physician–patient relationship and lack of time or palliative care services. Furthermore, physicians support use of the SQ, which could be used more routinely as a means to identify patients in their last phase of life, rather than accurately predicting death. Future studies should explore patients’ preferences for disclosing of their last phase of life.

## supplementary material

10.1136/bmjspcare-2020-002764 online supplemental file 1

## Data Availability

Data are available upon reasonable request. The focus group transcripts in Dutch that were analysed in this study are available from the corresponding author on reasonable request.
