# Supplementary material for: Physicians’ views on the usefulness and feasibility of identifying and disclosing patients’ last phase of life: a focus group study
Source: BMJ Support Palliat Care. 2021 Feb 22;14(e2):e002764. doi: 10.1136/bmjspcare-2020-002764 (PMC11671977; doi:10.1136/bmjspcare-2020-002764)
Supplement: online supplemental file 1 [file bmjspcare-14-e2-s001.pdf]

**Supplementary File: Coding tree**

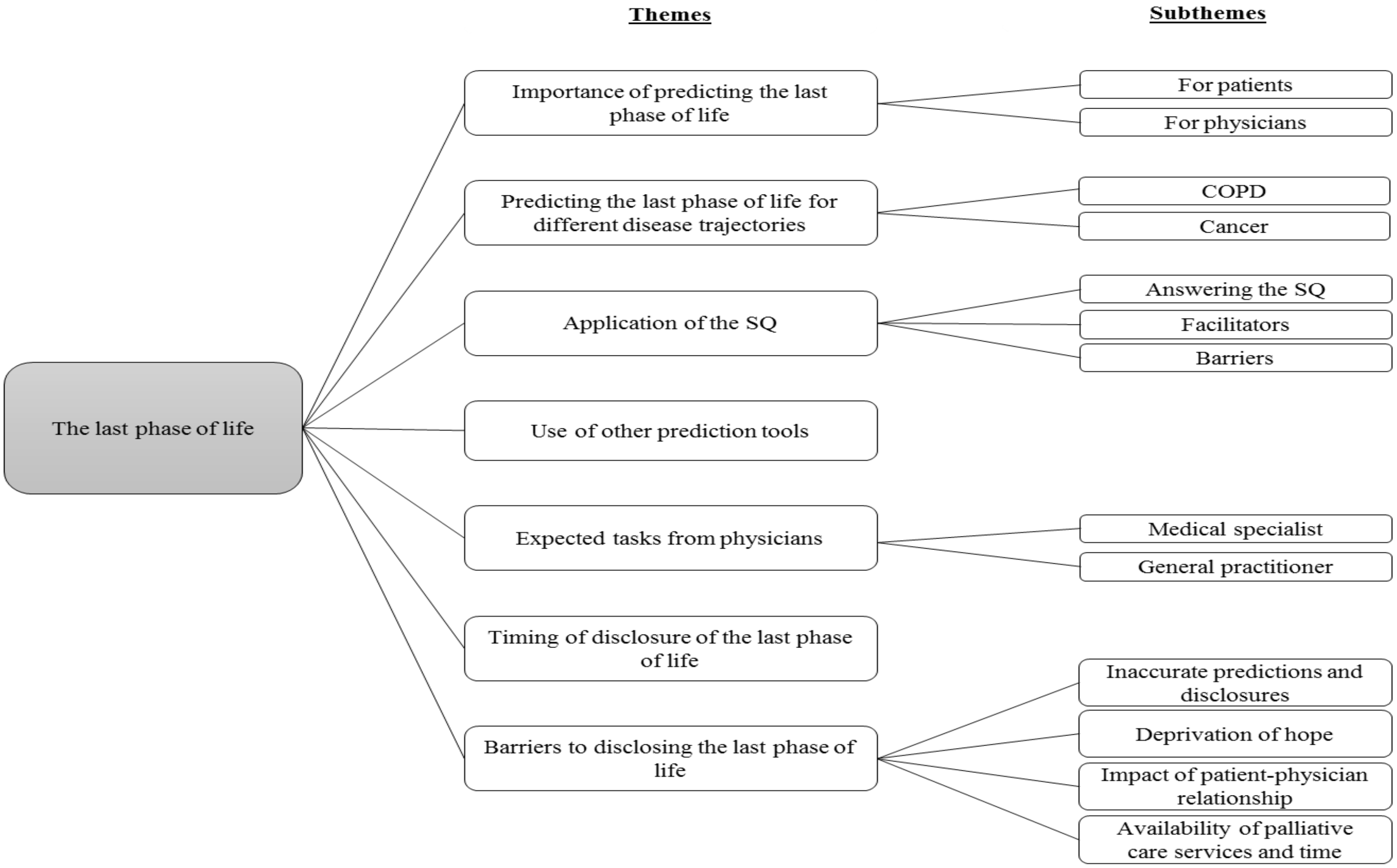


*COPD: Chronic Obstructive Pulmonary Disease; SQ: Surprise Question.*
